# Mass Cytometry Workflow to Achieve High‐Dimensional Immunophenotyping in Resource‐Limited or Decentralized Environments

**DOI:** 10.1002/cpz1.70335

**Published:** 2026-02-28

**Authors:** Natalie Smith, Michael Cohen, Lauren Tracey, Julie Alipaz, Christina Loh, David King, Neha Pulyani, Rebecca Auzins, Elin Gray, Sandra Taylor, Rajat Rai, Steven Kao, Barbara Fazekas de St Groth, Helen McGuire

**Affiliations:** ^1^ School of Medical Sciences, Faculty of Medicine and Health The University of Sydney Camperdown NSW Australia; ^2^ Standard BioTools South San Francisco California USA; ^3^ Edith Cowen University Perth Western Australia Australia; ^4^ Dubbo Base Hospital Dubbo New South Wales Australia; ^5^ Chris O'Brien Lifehouse Sydney New South Wales Australia

**Keywords:** CyTOF, immunophenotyping, mass cytometry

## Abstract

Globally, regional and remote communities are burdened by both an increased prevalence and worse prognosis of many infectious and chronic diseases. However, largely owing to logistical challenges, these communities are under‐represented in clinical trials and research studies. As individuals from rural communities experience unique environmental exposures and risk factors for disease, immune phenotyping data collected from metropolitan populations may not be broadly generalizable. To address this, we present a workflow that enables the inclusion of resource‐limited sites in high‐parameter mass cytometry studies. In this approach, whole blood (WB) or peripheral blood mononuclear cells (PBMCs) are collected, stained fresh for surface antigens, and cryopreserved at the collection site. Samples are then shipped to the central site for further processing, including neutrophil depletion, fixation, barcoding, intracellular staining, and data acquisition. Importantly, the WB staining approach does not require specialized equipment such as centrifuges and is therefore feasible to perform in a resource‐limited environment. A support protocol details steps for data preprocessing and cleanup. We present example data demonstrating the application of this workflow to determine immune differences between eight patients with late‐stage lung cancer and four healthy blood donors. Overall, this workflow may improve access to underserved communities and facilitate, for the first time, the scalability of immune phenotyping studies to harness geographically dispersed clinical centers. © 2026 The Author(s). *Current Protocols* published by Wiley Periodicals LLC.

**Basic Protocol 1**: Preparation and staining of PBMCs for cytometry

**Basic Protocol 2**: Preparation and staining of whole blood for cytometry

**Basic Protocol 3**: Fixation, permeabilization, intracellular staining, and data acquisition for blood sample immunophenotyping

**Support Protocol**: Data preprocessing and cleanup

## Introduction

Globally, regional and rural populations experience both a higher burden of disease and poorer health outcomes yet are rarely represented in biomedical research. This inequity is exemplified in the Australian context where, even though 30% of the population lives outside major cities, only 1.1% of national research funding between 2000 and 2014 was allocated to studying rural health (Barclay et al., 2018; Australian Institute of Health and Welfare, 2025). Current metropolitan‐centered biomedical research often relies on the availability of significant resources, specialized equipment, infrastructure, and staffing at each research site. Therefore, rural or resource‐limited sites, where specialized equipment such as centrifuges may not be available, are generally excluded from multi‐center studies designed for a metropolitan context.

Representation of rural or remote communities in biomedical research will be crucial to reduce inequities in disease burden. In fact, building a research culture around rural health services has been shown to improve both patient care and health outcomes (Paul et al., 2024; Schmidt et al., 2025). Rural communities live in a unique context, with exposure to diverse pathogens such as neglected tropical diseases (NTDs) and a higher incidence of many risk factors for chronic disease (Australian Institute of Health and Welfare, 2025; Kurcheid et al., 2022; MacIntyre et al., 2021). The environment we live in, and other non‐heritable influences, are known to drive variation in immune function between individuals (Brodin et al., 2015). Accordingly, results obtained from immune phenotyping of a metropolitan population may not be broadly generalizable to rural communities.

The application of cytometry in a rural or resource‐limited context is impeded by logistical challenges in sample preparation and storage. Because of its complexity, high‐dimensional cytometry is often best achieved with a centralized study design, in which samples are shipped to a central site for staining and data acquisition (Barsky et al., 2016; Le Lann et al., 2020; White et al., 2014). However, when blood samples must be shipped before they can be stabilized or cryopreserved, storage time and shipment temperature have significant effects on the quality of cells isolated (Jerram et al., 2021; Ng et al., 2012). This centralized approach is not feasible for truly remote communities, from which samples may not reach the central site within a reasonable time window, after which time their quality will have significantly degraded. However, a traditional decentralized study design is also not feasible in many resource‐limited communities in which standard laboratory equipment, or even electricity, may not be available. Reagents such as Cytodelics, Stable‐Lyse and Stable‐Store, Proteomic Stabilizer (PROT1), and TransFix may be used to quickly and easily stabilize blood at the collection site, overcoming some of these challenges (Gaudilliere et al., 2014; Rodriguez et al., 2020; Rybakowska et al., 2021). However, staining for several surface antigens, such as CCR6, CXCR3, CCR4, CCR7, CXCR5, and CD27, is not possible after stabilization as the fixatives present change binding epitopes (Dong et al., 2007; Nguyen et al., 2023; Sakkestad et al., 2020). Accordingly, in order to ensure resolution of subpopulation of T cells, staining must be performed at the collection site before stabilization.

To address these challenges, we have designed a mass cytometry workflow that enables the inclusion of remote communities in high‐parameter immune phenotyping studies. In this approach, blood samples are collected and stained fresh for surface antigens with dry‐format heavy‐metal conjugated antibodies. These dry‐format reagents, also referred to as the Maxpar Direct Immune Profiling Assay (MDIPA), allow reproducibility in staining signal to be achieved between technicians and across sites (Bagwell et al., 2020; Leipold et al., 2018). After staining, samples are cryopreserved without fixation until they can be shipped to a central site for further processing. We provide protocols for staining peripheral blood mononuclear cells (PBMCs) and whole blood (WB). Importantly, the WB staining approach can be implemented without the need for specialized equipment such as centrifuges at the collection site. This workflow is uniquely enabled by the stability of heavy‐metal‐conjugated antibodies, which allows stained samples to be cryopreserved and stored long‐term while maintaining antigen resolution (Schulz et al., 2019; Sumatoh et al., 2017). Overall, we demonstrate that approaches to high‐parameter mass cytometry can be simplified to make them compatible with resource limited settings while maintaining best‐quality data collection.

Basic Protocols 1 and 2 describe procedures for processing, surface staining, and cryopreservation of PBMCs and WB samples, respectively. Basic Protocol 2 includes additional instructions for neutrophil depletion from thawed WB samples. Basic Protocol 3 is designed to be performed after either previous protocol, and as such is compatible with both PBMC and neutrophil‐depleted WB sample types. It details fixation, permeabilization, barcoding, intracellular staining, and flow cytometric data acquisition for blood samples. The Support Protocol includes recommendations for data preprocessing and high‐dimensional analysis of samples.

## Strategic Planning

**Figure 1:**
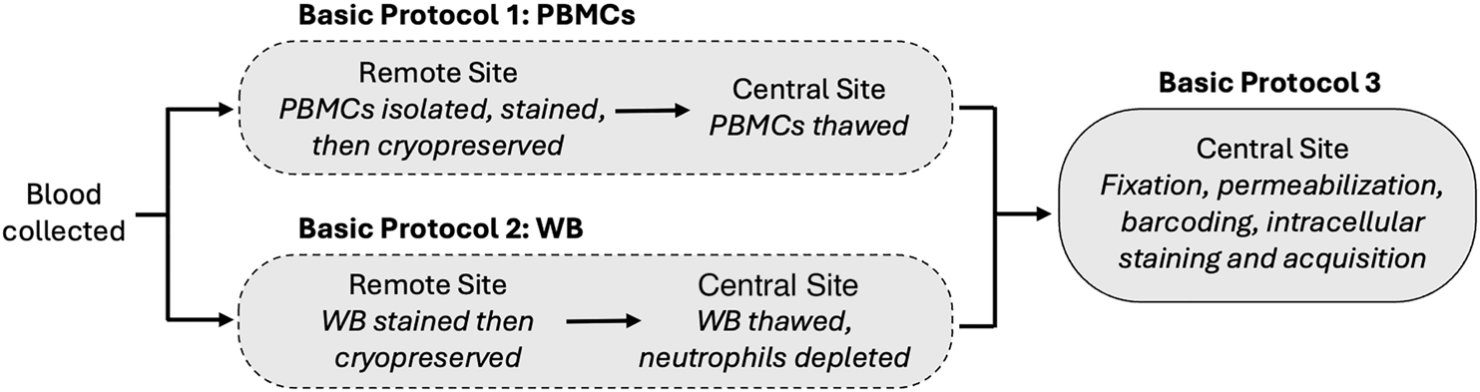
Overview of basic protocols. Basic Protocol 1 or 2 may be applied to stain peripheral blood mononuclear cells (PBMCs) or whole blood (WB), respectively. Basic Protocol 1 details the isolation of PBMCs, staining for surface antigens, and cryopreservation of PBMCs. Basic Protocol 2 requires access to no specialized equipment at the processing site and can therefore be applied in a resource‐limited setting. In this approach, whole blood samples are stained and cryopreserved at a remote site, and then neutrophils are depleted at the central site after samples are thawed. Basic Protocol 3 describes the fixation, permeabilization, barcoding, and intracellular staining of samples, as well as data acquisition through flow cytometry. It is compatible with both PBMCs (Basic Protocol 1) and neutrophil‐depleted WB (Basic Protocol 2).

Here, we present a mass cytometry approach that is applicable to a variety of settings. As summarized in Figure 1, either Basic Protocol 1 or 2 may be applied at the collection site to stain samples fresh for surface antigens. Basic Protocol 1 is best used in a setting where PBMCs are routinely isolated and cryopreserved. Basic Protocol 2 does not requires access to any specialized equipment at the processing site and is therefore suitable for application in resource‐limited settings. Studies designed to focus on PBMC phenotyping can benefit from neutrophil depletion from thawed WB samples at the central site. In this approach, removal of neutrophils reduces instrument acquisition time. These protocols enable a study design in which sites with different sample processing capabilities may be included concurrently. Correlate analysis of soluble circulating factors can be achieved by concurrent collection of plasma from the same blood draw.

All antibodies should be titrated to test a range of concentrations under the intended experimental conditions. An antibody concentration corresponding to the “plateau” of the curve should be selected to prevent minor experimental variation from affecting the signal intensity of antigen‐positive cells (Bonilla et al., 2024; Cossarizza et al., 2021). As WB is stained in the presence of neutrophils and in a larger volume than PBMCs, different concentrations of surface antibodies will be required for equivalent immune population resolution across the sample types. Plotting the positive signal of each antigen against the antibody concentration will produce a sigmoidal curve. After determination of optimal antibody working concentrations, antibody cocktails should be prepared in bulk and stabilized to ensure that all sites are working with the same reagents. Here, we apply a single‐test lyophilized format of the surface antibodies, which may be stored long term at 4°C. However, cocktails of mass cytometry antibodies may also be prepared in bulk and stored at −80°C in single‐test aliquots (Schulz et al., 2019).

To prevent intracellular ice crystal formation and ensure maximum cell viability after thaw, samples must be cryopreserved with a cryoprotective agent (dimethyl sulfoxide [DMSO]) at a cooling rate of ∼1°C per minute (Mazur, 1963). To achieve controlled‐rate cryopreservation, we use a CoolCell cryogenic freeze container (Corning); however, alternatives such as Mr. Frosty (Nalgene) may also be used. If the processing site lacks access to a freezer, controlled‐rate cryopreservation with dry ice should be optimized.

*CAUTION*: Sodium azide and paraformaldehyde (included in fixatives) are toxic and should be used with appropriate safety guidelines.

*NOTE*: Appropriate informed consent is necessary for obtaining and use of human study material.

## Basic Protocol 1 PREPARATION AND STAINING OF PBMCs FOR CYTOMETRY

The following protocol describes the steps to prepare and stain fresh PBMCs for surface antigens with lyophilized CyTOF antibodies. First plasma is removed from WB and then PBMCs are isolated. Three million PBMCs are removed, stained fresh for surface antigens with lyophilized CyTOF antibodies, and then cryopreserved and stored alongside any additional unstained PBMCs. After being shipped to a central site, the PBMCs are thawed and counted.

### Materials

EDTA‐anticoagulated whole peripheral blood (WB)
Phosphate‐buffered saline (PBS)/5% fetal bovine serum (FBS) (see recipe)
Ficoll‐Paque PLUS density‐gradient medium (Cytiva, cat. no. 17144003)
FACS buffer (see recipe)
Trypan blue
TruStain FcX (BioLegend, cat. no. 422302)
Lyophilized CyTOF antibodies (see Table 1)
1× freezing medium (see recipe)
Pierce Universal Nuclease for Cell Lysis (Thermo Fisher, cat. no. 88702)
RPMI/10% FBS (see recipe), prewarmed to 37°C

15‐ml centrifuge tubes
2‐ml screw‐cap micro tubes (cryovials), sterile (Sarstedt, cat. no. 72.694.005)
50‐ml centrifuge tubes
SepMate‐50 tubes (STEMCELL, cat. no. 85450)
Microplate
Hemocytometer
5‐ml round‐bottom tubes
CoolCell LX Freezing Container (Corning, cat. no. 432002)
−80°C freezer
Light microscope or automated cell counter

**Table 1:** Dry Format Antibody Panel

Isotype	Target	Clone
89 Y	CD45	HI30
103 Rh	Dead cells	Not applicable
111 Cd	CD33	WM53
112 Cd	HLA‐DR	L243
113 Cd	CD69	FN50
114 Cd	CD11b	ICRF44
115 In	CD11c	Bu15
141 Pr	CD3	UCHT1
142 Nd	CD19	HIB19
143 Nd	CD45RA	HI100
144 Nd	CCR5	HEK/1/85a
145 Nd	CD4	RPA‐T4
146 Nd	CD8a	RPA‐T8
147 Sm	CD20	2H7
148 Nd	CD16	3G8
149 Sm	CCR2	K036C2
150 Nd	CD86	IT2.2
151 Eu	CD39	A1
152 Sm	CD66b	G10F5
153 Eu	CD304	12C2
154 Sm	CCR6	11A9
155 Gd	CXCR5	RF8B2
156 Gd	PD‐1	EH12.2H7
158 Gd	CCR4	L291H4
159 Tb	CCR7	G043H7
160 Gd	CCR10	1B5
163 Dy	CD45RO	UCHL1
165 Ho	CD127	A019D5
166 Er	TIGIT	MBSA43
167 Er	CXCR3	G025H7
169 Tm	CD25	M‐A251
170 Er	CD123	6H6
172 Yb	CD38	HIT2
173 Yb	Integrin B7	FIB504
174 Yb	CD27	M‐T271
176 Yb	CD56	NCAM16.2
195 Pt	CD14	M5E2
196 Pt	IgD	IA6‐2

#### Isolate plasma (remote site)

1 Record the volume of blood collected. Centrifuge EDTA blood tubes for 15 min at 800 × *g*, room temperature.
  After centrifugation, three different fractions are distinguishable: the upper clear layer is plasma; the thin intermediate layer is buffy coat, containing concentrated leukocytes; and the bottom layer contains concentrated erythrocytes.
2 Use a sterile transfer pipet to remove the plasma.
  Remove as much plasma as possible without disturbing the buffy coat layer. Aim to leave ∼100 µl (~2 mm height in tube) of residual plasma behind.
3 Aliquot plasma into a 15‐ml tube and spin for 10 min at 1600 × *g*, room temperature.
4 Using a pipet and sterile tips, aliquot plasma into a maximum of four prelabeled sterile 2‐ml cryovials.
  When removing plasma, take care not to disturb the cell debris at the bottom of the tube or tubes.
5 Store plasma samples in −80°C freezer.

#### Isolate PBMCs (remote site)

6 Transfer plasma‐depleted blood to a fresh 50‐ml tube. Add a volume of prewarmed PBS/5% FBS equal to the original blood volume and mix gently.
7 Pipet 15 ml of Ficoll‐Paque PLUS density‐gradient medium into the central hole of the SepMate‐50 tube. Centrifuge SepMate‐50 tube for 1 min at 1000 × *g*, room temperature, to ensure removal of bubbles from the insert.
8 Slowly add the diluted blood to the SepMate tube by pipetting it down the side of the tube.
9 Centrifuge tubes for 20 min at 1200 × *g*, room temperature, with the brake switched ON.
  A clear layer of PBMCs should be visible. If this is not the case, increase centrifuge time to 30 min or change the brake to light.
10 After centrifugation, invert the tube (for no longer than 2 sec) and pour the PBMCs into a new labeled 15‐ml tube. Dilute to 15 ml with prewarmed PBS/5% FBS.
  After centrifugation, red blood cells and granulocytes will be under the SepMate‐50 insert, and PBMCs and plasma will remain on top of the insert. Ensure that red blood cells are not tipped out when removing PBMCs.
11 Wash PBMCs by centrifugation for 10 min at 300 × *g*, room temperature, and then tip off the supernatant into liquid waste. Repeat this wash step once more for a total of two washes or until the supernatant is clear.
  The washes are designed to remove platelets from PBMC isolates.
12 Resuspend PBMCs in 10 ml of FACS buffer. Remove 10 µl from the 10 ml of cell suspension and aliquot into a microplate for counting.
13 Combine the 10 µl of cell suspension with 10 µl of trypan blue, and then aliquot 10 µl of the mixture into a hemocytometer. Perform manual cell counting under a light microscope or use an automated cell counter. Record the number of viable cells. Note the presence of debris or red blood cells.
14 Calculate the required volume to remove 3 million cells from the 10 ml of cell suspension. Aliquot this volume into a labeled 5‐ml tube.

#### Stain PBMCs for surface antigens (remote site)

15 Centrifuge 5‐ml tube containing 3 million PBMCs for 5 min at 500 × *g*, room temperature, and then remove the supernatant.
16 Resuspend cells in residual volume and then add 5 µl of TruStain FcX to each 5‐ml tube. Gently vortex to mix.
17 Incubate for 15 min at room temperature.
18 During incubation, remove tube containing lyophilized CyTOF antibodies from the refrigerator and from its foil packaging and allow to equilibrate to room temperature.
19 Transfer the entire volume (∼70 µl) from the 5‐ml tube to the tube containing lyophilized CyTOF antibodies, ensuring that the lyophilized pellet is fully dissolved.
20 Gently vortex to mix each tube, and incubate the tubes at room temperature for 30 min.
21 During the incubation, finish preparing any remaining unstained PBMCs for cryopreservation by adding 30 ml of PBS/5% FBS to the 50‐ml Falcon tube containing the remaining unstained PBMCs. Centrifuge for 10 min at 300 × *g*, room temperature, and then tip off the supernatant. Cryopreserve unstained PBMCs at 2‐4 million cells per ml of 1× freezing medium in a CoolCell.
  Be sure to clearly label vials as “stained” or “unstained.”
22 After the cell staining incubation (step 20), wash by adding 2 ml FACS buffer to each 5‐ml tube, centrifuge for 5 min at 500 × *g*, room temperature, and remove supernatant. Repeat for a total of two washes.
23 Resuspend stained cells in 1 ml of 1× freezing medium and then transfer the full volume to a cryovial labeled with “stained PBMCs,” donor ID, and date.
24 Place cryovials of PBMCs in a CoolCell in a −80°C freezer. Move to liquid nitrogen for long‐term storage.
  If performing a multi‐site study, samples may be collected longitudinally and sent to the central site in batches.

#### Thaw PBMCs (central site)

25 Retrieve cryovials from frozen storage. Rapidly thaw cryovials in a 37°C water bath. Transfer thawed PBMCs to a 15‐ml tube containing 10 ml of prewarmed (37°C) RPMI/10% FBS, supplemented with 1 µl Pierce Universal Nuclease.
26 Centrifuge suspension for 8 min at 350 × *g*, room temperature, and discard supernatant. Resuspend in 1 ml of RPMI/10% FBS and transfer to a 5‐ml tube.
27 Remove 10 µl from the cell suspension and aliquot into a microplate. Leave the remaining 1 ml of cell suspension at room temperature while counting the cells.
28 Combine the 10 µl of cell suspension with 10 µl trypan blue, and then aliquot 10 µl of the mixture into a hemocytometer. Perform manual cell counting under a light microscope or with an automated cell counter and record the number of viable cells. Note the presence of debris in the sample.
29 Centrifuge 5‐ml tube containing cell suspension for 5 min at 500 × *g*, room temperature, and discard the supernatant. Continue to Basic Protocol 3.

## Basic Protocol 2 PREPARATION AND STAINING OF WHOLE BLOOD FOR CYTOMETRY

The following protocol describes the steps to prepare and stain fresh WB for surface antigens with lyophilized CyTOF antibodies. This protocol does not require centrifugation and is compatible with a resource‐limited setting. Briefly, plasma is removed from WB, and then 500 µl of plasma‐depleted WB is added directly to the lyophilized CyTOF antibodies. Stained WB is cryopreserved directly without washes. At the central lab, WB is thawed in batches and neutrophils are depleted with magnetic beads.

### Materials

Peripheral blood collected in EDTA‐coated tubes
TruStain FcX (BioLegend, cat. no. 422302)
Lyophilized CyTOF antibodies (see Table 1)
2× freezing medium (see recipe)
RPMI/10% FBS (see recipe), prewarmed to 37°C
Pierce Universal Nuclease for Cell Lysis (Thermo Fisher, cat. no. 88702)
FACS buffer (see recipe)
Anti‐CD15 Biotin (BioLegend, cat. no. 301914)
Anti‐Biotin MicroBeads (Miltenyi Biotec, cat. no. 130‐090‐485)
Trypan blue

Transfer pipets, sterile
15‐ml centrifuge tubes
5‐ml round‐bottom tubes
2‐ml screw‐cap micro tubes (cryovials), sterile (Sarstedt, cat. no. 72.694.005)
−80°C freezer or dry ice
CoolCell LX Freezing Container (Corning, cat. no. 432002)
Liquid nitrogen
LS Columns (Miltenyi Biotec, cat. no. 130‐042‐401)
QuadroMACS Separator (Miltenyi Biotec, cat. no. 130‐091‐051)
Microplate
Hemocytometer
Light microscope or automated cell counter

#### Isolate plasma (remote site)

1 Record the volume of blood collected. Centrifuge EDTA blood tubes for 15 min at 800 × *g*, room temperature.
  After centrifugation, three different fractions are distinguishable: the upper clear layer is plasma; the thin intermediate layer is buffy coat, containing concentrated leukocytes; and the bottom layer contains concentrated erythrocytes. If a centrifuge is not available at the processing site, leave blood to settle at room temperature.
2 Use a sterile transfer pipet to remove the plasma.
  Remove as much plasma as possible without disturbing the buffy coat layer. Aim to leave ∼100 µl (1‐2 mm) of residual plasma behind.
3 Aliquot plasma into a 15‐ml tube and spin for 10 min at 1600 × *g*, room temperature.
  Skip this step if centrifuge is not available at the processing site.
4 Using a pipet and sterile tips, aliquot plasma into a maximum of four prelabeled sterile 2‐ml cryovials. Ensure that the first vial contains 1.5‐2 ml plasma, and then evenly aliquot the rest of the plasma across the remaining cryovials.
  When removing plasma, take care not to disturb the cell debris at the bottom of the tube/s.
5 Store plasma samples in −80°C freezer.

#### Stain WB for surface antigens (remote site)

6 Transfer 500 µl of plasma‐depleted blood from step 2 to a 5‐ml round‐bottom tube. Add 5 µl of TruStain FcX and mix well.
7 Incubate for 15 min at room temperature.
8 During the incubation, remove the tube of lyophilized antibody from the refrigerator and from within its foil packaging and allow to equilibrate to room temperature.
9 Transfer the full volume from the 5‐ml tube to the tube of lyophilized antibody and ensure that the lyophilized pellet is fully dissolved.
10 Gently mix the contents of each tube and incubate the tubes at room temperature for 30 min.
11 Add 500 µl of 2× freezing medium to each tube of stained blood (to a final concentration of 1× freezing medium in 1 ml), and then transfer the full volume to a cryovial labeled “stained WB.”
  Unstained WB may also be cryopreserved in at a 1:1 ratio in 2× freezing medium.
12 Place cryovials of PBMCs in a CoolCell in a −80°C freezer. Move to liquid nitrogen for long‐term storage.

#### Thaw WB and deplete neutrophils (central site)

13 Retrieve cryovials of stained WB from frozen storage. Rapidly thaw cryovials in a 37°C water bath. Transfer thawed WB to a 15‐ml tube containing 10 ml prewarmed RPMI/10% FBS, supplemented with 1 µl Pierce Universal Nuclease.
14 Centrifuge suspension for 8 min at 350 × *g*, room temperature, and discard supernatant. Resuspend in 1 ml of RPMI/10% FBS and transfer to a 5‐ml tube.
15 Add 100 µl of Anti‐CD15 Biotin diluted 1:1000 in FACS buffer.
16 Incubate for 5 min on ice.
17 Wash by adding 1 ml FACS buffer to each 5‐ml tube, centrifuging for 5 min at 500 × *g*, 4°C, and removing supernatant. Repeat for a total of two washes.
18 Resuspend WB in 70 µl of FACS buffer and then add 20 µl of Anti‐Biotin MicroBeads.
19 Incubate for 15 min on ice.
20 Wash by adding 1 ml FACS buffer to each 5‐ml tube, centrifuging for 5 min at 500 × *g*, 4°C, and removing supernatant. Repeat for a total of two washes.
21 Resuspend samples in 500 µl of FACS buffer.
22 Place LS Columns in the magnetic field of a QuadroMACS Separator. Rinse columns with 3 ml of FACS Buffer.
23 Apply cells to the LS Column and collect neutrophil‐depleted flow‐through in a 15‐ml tube. Wash column three times with 3 ml of FACS buffer each time.
24 Remove column from the separator, add 5 ml of FACS buffer, and flush out magnetically labeled neutrophils by pushing the plunger into the column. Discard isolated neutrophils.
25 Remove 10 µl from the neutrophil‐depleted WB and aliquot into a microplate well.
26 Combine the 10 µl of cell suspension with 10 µl trypan blue, and aliquot 10 µl of the mixture into a hemocytometer. Perform manual cell counting under a light microscope or with an automated cell counter and record the number of viable cells.
27 Centrifuge 15‐ml tube containing neutrophil‐depleted WB for 8 min at 350 × *g*, room temperature, and discard the supernatant. Resuspend cells in 1 ml FACS buffer and transfer the full volume to a 5‐ml tube. Continue with Basic Protocol 3.

## Basic Protocol 3 FIXATION, PERMEABILIZATION, INTRACELLULAR STAINING, AND DATA ACQUISITION FOR BLOOD SAMPLE IMMUNOPHENOTYPING

The following protocol is designed to follow directly from both Basic Protocol 1 and Basic Protocol 2. It details barcoding, staining for intracellular antigens, and acquisition of sample data using a CyTOF system. Briefly, stained PBMCs or neutrophil‐depleted WB are fixed and permeabilized. Cells are then barcoded and multiplexed. When analyzing longitudinal samples from the same donor, these should be stained with different barcodes and combined. After barcoding, cells are stained for surface antigens then for DNA content. Samples may either be acquired within 48 hr of staining or cryopreserved for longer‐term storage before acquisition.

### Materials

Foxp3/Transcription Factor Staining Buffer Set (eBioscience, cat. no. 00‐5523‐00)
Cell‐ID 20‐Plex Pd Barcoding Kit (Standard BioTools, cat. no. 201060)
CyTOF antibodies for intracellular targets (see Table 2)
FACS buffer (see recipe)
Maxpar Cell Acquisition Solution (Standard BioTools, cat no. 201240)

5‐ml round‐bottom tubes
15‐ or 50‐ml centrifuge tubes as needed (see step 10)
Ultrafree Centrifugal Filter (Millipore, cat. no. UFC30DV25)
2‐ml screw‐cap micro tubes (cryovials), sterile (Sarstedt, cat. no. 72.694.005)
CoolCell LX Freezing Container (Corning, cat. no. 432002)
−80°C freezer
5‐ml Falcon Round‐Bottom Tubes with Cell Strainer Cap (STEMCELL, cat. no. 38030)
EQ Six Element Calibration Beads (100 ml; Standard BioTools, cat. no. 201245)
CyTOF instrument: e.g., Helios system with standard equipment and CyTOF software

**Table 2:** Antibodies for Intracellular Targets

Isotype	Target	Clone
161 Dy	CD247	6B10.2
162 Dy	FoxP3	PCH101
164 Dy	Eomes	WD1928
168 Er	Ki67	B56
171 Yb	Arginase I	14D2C43
175 Lu	Perforin	B‐D48
198 Pt	Granzyme B	GB11
209 Bi	T‐bet	4B10

#### Fixation/permeabilization (central site)

1 Prepare fixation/permeabilization working solution by mixing one part Foxp3 Fixation/Permeabilization Concentrate with three parts Foxp3 Fixation/Permeabilization Diluent (both from the Foxp3/Transcription Factor Staining Buffer Set). One milliliter of the working solution is required for each sample.
2 Add 1 ml of fixation/permeabilization working solution to each 5‐ml tube and gently pipet to mix. Incubate at room temperature for 30 min.
3 Wash by adding 1 ml of 1× Permeabilization Buffer (from the Foxp3/Transcription Factor Staining Buffer Set) to each 5‐ml tube, centrifuging for 5 min at 900 × *g*, 4°C, and removing supernatant. Repeat for a total of two washes.
  The increased centrifuge speed after cell fixation results in greater cell recovery.
  If not performing barcoding, skip to step 14 for intracellular staining protocol. Otherwise, proceed with steps 4‐13 first.

#### Barcoding

4 Before beginning the protocol, obtain the necessary number of barcodes from the Cell‐ID 20‐Plex Pd Barcoding Kit. One unique barcode is required per sample. Return the remaining kit components to the −20°C freezer. Allow 10 min for the barcodes to come to room temperature, and then briefly centrifuge to ensure that all liquid reaches the bottom of the tube.
5 Resuspend each sample to be barcoded in 800 µl of 1× Permeabilization Buffer.
  It is important to disrupt the cell pellet and thoroughly mix for uniform barcode staining.
6 Resuspend each Cell‐ID barcode in 100 µl of 1× Permeabilization Buffer and transfer to the appropriate sample. Immediately mix the samples.
7 Incubate samples at room temperature for 30 min. After 15 min, gently vortex each tube to mix.
8 Wash by adding 1 ml of 1× Permeabilization Buffer to each 5‐ml tube, centrifuging for 5 min at 900 × *g*, 4°C, and removing supernatant. Repeat for a total of two washes.
9 Add 1 ml of 1× Permeabilization Buffer to each sample.
10 Combine all uniquely barcoded samples into one tube. A maximum of 20 samples may be combined when using the Cell‐ID 20‐Plex Pd Barcoding Kit. Transfer to tubes of the required size, depending on the number of cells being stained.
  a. 1‐3 million cells: 2‐ml tubes
  b. 3‐15 million cells: 5‐ml tubes
  c. 15‐45 million cells: 15‐ml tubes
  d. 45‐60 million cells: 50‐ml tubes
11 Rinse each individual barcoded sample tube two more times with 100 µl of 1× Permeabilization Buffer and transfer to the combined tube to maximize cell recovery.
12 Reserve a small volume (∼10 µl) from the combined tube to count cells in the multiplexed sample to ensure optimal antibody staining.
13 Centrifuge cells for 5 min at 900 × *g*, 4°C, carefully aspirate supernatant, and gently vortex to resuspend cells in the residual volume.
  Proceed with steps 1‐21, adjusting the antibody scale, staining, and washing volumes to accommodate the number of cells in the multiplexed sample.

#### Intracellular staining and Cell‐ID Intercalator‐Ir

14 If continuing from step 3, gently vortex tube to resuspend cells in residual volume.
15 Prepare 100 µl of intracellular antibody cocktail per 3 million cells in 1× Permeabilization Buffer.
  Antibody cocktails can be made in bulk and stored at −80°C. Briefly, aliquot all required volume of antibodies to a tube with 10% excess volume. Mix well and make individual aliquots, and then seal and freeze at −80°C.
16 Filter intracellular antibody cocktail through an Ultrafree Centrifugal Filter for 3 min at 12,000 × *g*, 4°C.
  This ensures the removal of antibody aggregates.
17 Add the 100 µl of intracellular antibody cocktail per 3 million cells and incubate for 30 min at 4°C.
18 Add 1 ml of 1× Permeabilization Buffer per 3 million cells to each tube. Centrifuge samples for 5 min at 900 × *g*, 4°C. Discard the supernatant.
19 Repeat washes in 1× Permeabilization Buffer twice more and resuspend in residual volume, ensuring pellet is fully disrupted and mixed thoroughly.
20 Prepare intercalation solution (see recipe) and add 1 ml per 3 million cells. Vortex samples to ensure pellet is fully dissolved. Refrigerate samples in intercalation solution overnight.
  Samples can be stored in intercalation solution at 2‐8°C for up to 48 hr before sample acquisition. If not acquiring data within 48 hr, samples can be frozen and stored at −80°C as below.
21 *Optional*: Centrifuge samples for 5 min at 900 × *g*, 4°C. Remove supernatant and resuspend cells in 1× freezing medium. Place cryovials of PBMCs in a CoolCell in a −80°C freezer and store until acquisition.

#### Data acquisition

22 If samples were cryopreserved before acquisition, thaw at 4°C and then wash in 1 ml Milli‐Q water with for 5 min with centrifugation 900 × *g*, 4°C. If samples were not cryopreserved, first wash with 1 ml FACS buffer and then continue with Milli‐Q water wash as above. Remove supernatant and resuspend in 1 ml Maxpar Cell Acquisition Solution.
23 Remove 10 µl from the suspension and count.
24 Centrifuge for 5 min at 900 × *g*, 4°C, remove supernatant, and resuspend in the appropriate volume of Maxpar Cell Acquisition Solution containing 1:10 EQ Six Element Calibration Beads to obtain a concentration of 800,000 cells/ml. Run the sample through a 35‐µm Cell Strainer Cap to remove clumps.
25 Acquire sample data on a CyTOF instrument.
  Acquire samples to completion at 30 µl/min and ≤450 events/s.

## DATA PREPROCESSING AND CLEANUP

Here, we describe the steps required to preprocess CyTOF files for analysis. First, samples are normalized with the concurrently run EQ Beads using the CyTOF acquisition software. Normalized .fcs files are then imported into an analysis software (such as FlowJo), where “cleanup” gating is performed by removing beads, selecting live single cells, and then debarcoding files based on their palladium barcode signal. Pre‐processed files are then exported from the analysis software for subsequent high‐dimensional analysis. In this protocol, we show representative results from dimension reduction and clustering to demonstrate visualization and interpretation of high‐dimensional data.

### Materials

Software for analysis: e.g., FlowJo or CellEngine
.fcs files acquired in CyTOF software (see Basic Protocol 3)

#### Data normalization and debarcoding on the CyTOF Software

1 Under the processing tab in the CyTOF software, launch the FCS Processing tool and select .fcs files to normalize.
  This will normalize files to the signal from the concurrently run EQ beads and create a new .fcs file.
2 Under the processing tab in CyTOF, launch the Debarcoder tool and click Barcode Manager. Open the barcode key file and enter unique sample names corresponding to each barcode in the Sample columns. Uncheck any barcodes that were not used in the sample. Click SaveAs to save the resulting barcode key with a unique name.
  The file indicates the masses of the six Pd isotopes included in the 20‐plex kit. The Output Path represents the names of the files that will be generated when debarcoding is completed.
3 Click Open to browse for the normalized file, and then click on Debarcode. In the Separation tab, check the barcode separation plots and choose a BcS value just before the event yield drops significantly. This chosen BcS value will be displayed as a vertical dotted red line on the plots.
4 Click Save Debarcoded Files to generate a new .fcs file for each debarcoded sample.

#### Sample preprocessing and cleanup

**Figure 2:**
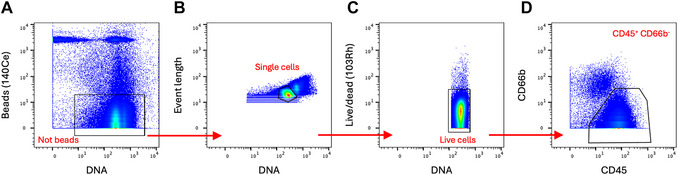
Gating tree for data preprocessing and cleanup. Dotplots exported from FlowJo demonstrate the gating hierarchy for data cleanup, with plots annotated with the gate name. Biaxial dotplots show the results of gating for (**A**) 140Ce and DNA to remove beads, (**B**) event length and DNA to remove doublet cells, (**C**) 103Rh and DNA to remove dead cells, and (**D**) CD66b and CD45 to select CD45^+^ non‐neutrophils.

5 Upload normalized and debarcoded files to an appropriate analysis software package, such as FlowJo.
6 Create two‐dimensional plot of 140Ce and DNA to select cells and remove beads (Fig. 2A).

**Figure 3:**
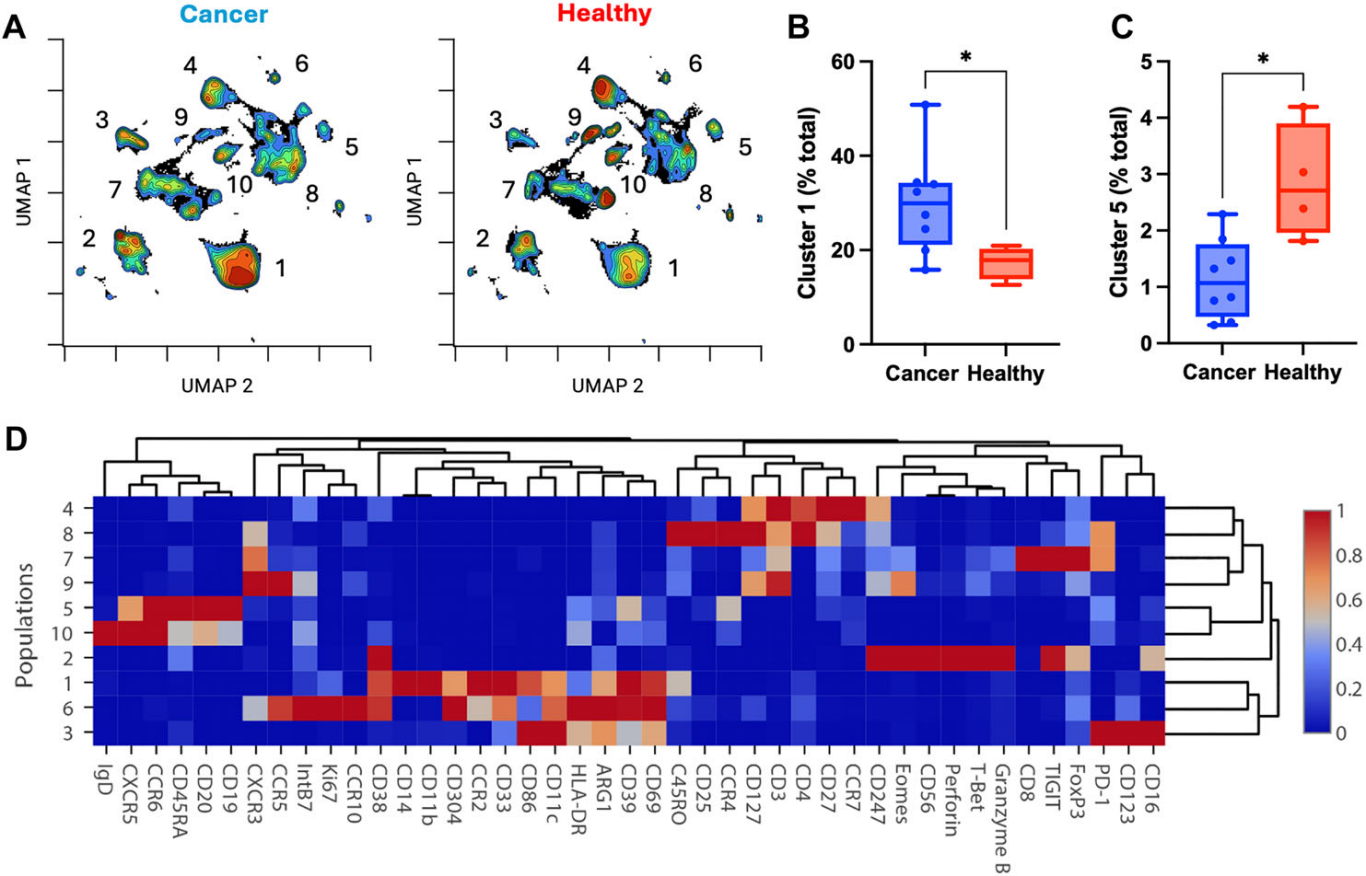
High‐dimensional immune profiling of cancer patients and healthy donors. Peripheral blood mononuclear cells (PBMCs) from eight patients with lung cancer and four healthy blood donors were isolated, stained, and subjected to flow cytometry as described in Basic Protocol 1 and Basic Protocol 3. Acquired sample data were normalized, debarcoded, and cleanup gated as described in the Support Protocol. Cleaned files were uploaded to CellEngine and downsampled to an equivalent number of events. (**A**) Uniform manifold approximation and projection (UMAP) dimensionality reduction was performed using all antigens as analysis channels. Ten major clusters were gated, with cluster ID annotated on representative UMAP plots from one cancer patient and one healthy donor. (**B**, **C**) Bar charts with Mann‐Whitney tests comparing abundance of (**B**) cluster 5 and (**C**) cluster 1 between cancer patients and healthy donors. *p ≤ .05; **p ≤ .01. (**D**) Heatmap of scaled mean signal intensity of each antigen in the 10 major clusters generated in CellEngine. Average linkage clustering performed on both populations and channels.

7 Create two‐dimensional plot of Event Length and DNA to remove doublet cells (Fig. 2B).
8 Create two‐dimensional plot of 103Rh and DNA to remove dead cells (Fig. 2C).
9 Create two‐dimensional plot of CD66b and CD45 to remove neutrophils and select CD45^+^ events (Fig. 2D).
10 Export cleaned files for high‐dimensional analysis (Fig. 3).

## Reagents and Solutions

### FACS buffer

1000 ml of 1× phosphate‐buffered saline (PBS; Sigma‐Aldrich, cat. no. P4417‐50TAB)
2 ml of 0.1 mM EDTA, pH 8.0, RNase‐free (ThermoFisher, cat. no. AM9912)
5 g bovine serum albumin (BSA; Merck, cat. no. A7906‐100g)
2 ml of 10% (w/v) sodium azide (Chem Supply Australia, cat. no. 3902)
Filter through a 22‐µm‐pore‐size filter (Corning, cat. no. CLS431097‐1EA)
Store up to 1 month at 4°C

### Freezing medium, 1×

20 ml heat‐inactivated fetal bovine serum (FBS; Sigma‐Aldrich, cat. no. F9423‐500ML)
20 ml RPMI 1640 medium (ThermoFisher, cat. no. 11875119)
10 ml DMSO (AJAX FineChem, cat. no. AJA2225‐500)
Store up to 1 month at 4°C

### Freezing medium, 2×

20 ml heat‐inactivated FBS (Sigma‐Aldrich, cat. no. F9423‐500ML)
10 ml RPMI 1640 Medium (ThermoFisher, cat. no. 11875119)
20 ml DMSO (AJAX FineChem, cat. no. AJA2225‐500)
Store up to 1 month at 4°C

### Intercalation solution

1 µl of 500 µM Cell‐ID Intercalator‐Ir (Standard BioTools, cat. no. 201192B)
1 ml of 16% (w/v) aqueous paraformaldehyde, methanol free (ThermoFisher, cat. no. 043368‐9M)
3 ml of 1× PBS (Sigma‐Aldrich, cat. no. P4417‐50TAB)
Scale to prepare 1 ml per sample. Prepare fresh for each experiment.

### PBS/5% FBS

475 ml of 1× PBS (Sigma‐Aldrich, cat. no. P4417‐50TAB)
25 ml heat‐inactivated FBS (Sigma‐Aldrich, cat. no. F9423‐500ML)
Filter through a 22‐µm‐pore‐size filter (Corning, cat. no. CLS431097‐1EA)
Store up to 1 month at 4°C

### RPMI/10% FBS

450 ml RPMI 1640 medium (ThermoFisher, cat. no. 11875119)
50 ml heat‐inactivated FBS (Sigma‐Aldrich, cat. no. F9423‐500ML)
Filter through a 22‐µm‐pore‐size filter (Corning, cat. no. CLS431097‐1EA)
Store up to 1 month at 4°C

## Commentary

### Critical Parameters and Troubleshooting

Antibody titration is an essential first step in any cytometric workflow. All antibodies should be titrated with representative experimental conditions, including number of cells stained, staining time, and staining volume. When designing panels for mass cytometry, weakly expressed antigens should be assigned channels in the 153‐176 range, where the CyTOF instrument is most sensitive. Different anticoagulants are routinely used in blood collection tubes in the clinical setting. Although we use EDTA‐coated collection tubes, this anticoagulant may interfere with downstream functional assays (Browne et al., 2024; Kumar & Satchidanandam, 2000). If planning to perform functional assays alongside immune cell phenotyping, heparinized collection tubes should be used. Blood samples should be kept at room temperature after collection and should be processed within 8 hr to ensure best resolution of surface antigens (Sedek et al., 2022). Prolonged time from collection to processing will cause a significant loss of sample quality with increased neutrophil contamination in PBMCs, reduced sample yield, and reduced viability (Jerram et al., 2021; Yi et al., 2023). The time of blood collection, time of processing, and staining time should be recorded for each sample to assess the impact of these variables on downstream immune phenotype data. We found that staining 2‐3 million PBMCs or 500 µl of plasma‐depleted WB yielded sufficient cells after acquisition. However, the number of PBMCs or volume of WB stained may need to be adjusted depending on the aims of each experiment. Staining conditions such as volume, time, and temperature should be kept consistent across all samples. If performing WB staining, ensure that CD15 is not included in the staining cocktail to enable neutrophil depletion with anti‐CD15 post‐sample thawing.

Here, we describe the use of the eBioscience Foxp3/Transcription Factor Staining Buffer Set before intracellular staining. However, different fixatives or permeabilizing agents may be appropriate if not performing staining for transcription factors or nuclear antigens. The wash steps performed after intracellular staining are essential to reduce background staining and ensure best resolution of intracellular antigens. Previous studies to optimize the cryopreservation of mass cytometry stained samples only tested the effects of cryopreservation for up to 4 weeks (Sumatoh et al., 2017). However, we have cryopreserved samples stained with the panel presented for up to 9 months with no detectable loss of signal intensity. The stability of staining signal after cryopreservation should be determined for each antigen. Samples must undergo a second fixation step in 4% PFA overnight to ensure that cells are not lysed when resuspended in water before data acquisition. If significant cell loss is observed after washing cells in water, it is possible that they were not sufficiently fixed. Sample data should be acquired in large‐scale batches with a batch control in order to account for technical batch effects. As data are acquired during mass cytometry at an event rate of 250‐500 events/s, it may not be logistically possible to acquire all large samples to completion. Mass cytometers have a transmission efficiency of ∼50%, meaning roughly half of the cells in suspension before acquisition will be recorded as events (Olsen et al., 2019). The number of events that should be acquired per sample is dependent on the aims of each individual experiment. If you are aiming to detect and measure rare cell populations, it may be necessary to acquire more cells to ensure that detection limits are reached (Soh & Wallace, 2019).

Barium (Ba) is present in many laboratory soaps and may cause contamination of 130 Ba to 138 Ba channels during acquisition (Leipold et al., 2015). This should be avoided even if these channels are not used within the experiment as it can cause aging of the detector. In general, reagents for mass cytometry should be prepared and stored in plastic vessels that have never been washed. Lead (Pb) contamination may also be found in laboratory buffers. To determine the sources of metal contaminants, a diluted aliquot of the suspected reagent should be acquired on the mass cytometer and observed.

### Understanding Results

Data analysis steps will vary significantly depending on each experimental design and study aims. Approaches to the analysis of mass cytometry data have been reviewed in great detail elsewhere (Kimball et al., 2018; Pedersen & Olsen, 2019). Here, we present example data obtained to compare the phenotype of PBMCs from late‐stage lung cancer patients and healthy donors. Briefly, PBMCs were isolated, stained fresh for surface antigens, and then cryopreserved at two sites. Samples were shipped to the central site where fixation, barcoding, and intracellular staining were performed. After following the data preprocessing and cleanup steps detailed in the Support Protocol, files were uploaded to CellEngine for high‐dimensional analysis. Samples were downsampled to an equivalent number of events and uniform manifold approximation, and projection (UMAP) analysis was performed on the samples, using all antigens as analysis channels (Fig. 3A). Gates were manually drawn around the clusters in the UMAP, and they were annotated with their cluster ID. The frequencies of each cluster within each sample were exported from CellEngine and assessed between the groups. The abundance of cluster 1 was significantly higher in cancer patients than in healthy controls (*p* = .0485; Fig. 3B). Additionally, cluster 5 was significantly less abundant in PBMCs from cancer patients (*p* = .0162). To determine the immune populations represented by each cluster, a heatmap of antigen expression across the clusters was generated (Fig. 3C). By assessment of antigen expression, cluster 1 was identified as monocytes (HLA‐DR^+^ CD14^+^ CD11b^+^) and cluster 5 as memory B cells (CD19^+^ CD20^+^ IgD^−^). Accordingly, monocytes are more abundant and memory B cells less abundant in late‐stage lung cancer patients compared to healthy individuals. Manual gating should be performed alongside any high‐dimensional analysis to validate findings. Additionally, the mean signal intensity of each antigen on each cell population may be assessed to determine phenotypic differences between study cohorts. Previous studies by our group detail additional examples of the application of mass cytometry to study a range of pathologies, including coronary artery disease, cytomegalovirus in stem cell recipients, and adverse events from checkpoint immunotherapy (Kott et al., 2023; McGuire et al., 2018, 2020).

### Time Considerations

“Remote site” steps 1‐24 of Basic Protocol 1 take ∼2‐2.5 hr to perform, with PBMC processing being the most time‐consuming step. “Remote site” steps 1‐12 of Basic Protocol 2 take ∼1 hr to perform. The Support Protocol takes ∼1 hr to perform.

### Author Contributions

**Natalie Smith**: Conceptualization; data curation; formal analysis; funding acquisition; investigation; methodology; project administration; visualization; writing—original draft; writing—review and editing. **Michael Cohen**: Methodology; resources; writing—review and editing. **Lauren Tracey**: Methodology; resources; writing—review and editing. **Julie Alipaz**: Project administration. **Christina Loh**: Methodology; resources; writing—review and editing; project administration. **David King**: Methodology; resources; writing—review and editing; project administration. **Neha Pulyani**: Investigation. **Rebecca Auzins**: Investigation. **Elin Gray**: Investigation; supervision. **Sandra Taylor**: Resources; project administration. **Rajat Rai**: Resources; project administration. **Steven Kao**: Resources; project administration. **Barbara Fazekas de St Groth**: Funding acquisition; supervision; writing—review and editing. **Helen McGuire**: Conceptualization; funding acquisition; project administration; resources; supervision; writing—review and editing.

### Conflict of Interest

In‐kind support for reagents was provided by Standard BioTools.

## Data Availability

The data, tools, and materials (or their sources) that support the protocol are available from the corresponding author upon reasonable request.
